# Cysteamine bitartrate delayed‐release capsules control leukocyte cystine levels and promote statural growth and kidney health in an open‐label study of treatment‐naïve patients <6 years of age with nephropathic cystinosis

**DOI:** 10.1002/jmd2.12260

**Published:** 2021-11-01

**Authors:** Maria Helena Vaisbich, Juliana Caires Ferreira, Heather Price, Kyleen D. Young, Saba Sile, Gregg Checani, Craig B. Langman

**Affiliations:** ^1^ São Paulo University Medical School São Paulo Brazil; ^2^ Instituto da Criança do Hospital das Clínicas da Faculdade de Medicina da Universidade de São Paulo (HCFMUSP) São Paulo Brazil; ^3^ Ann & Robert H. Lurie Children's Hospital of Chicago Chicago Illinois USA; ^4^ Horizon Therapeutics plc Deerfield Illinois USA; ^5^ Feinberg School of Medicine Northwestern University Chicago Illinois USA

**Keywords:** anthropometric parameters, children, cysteine, cystinosis, delayed‐release cysteamine, immediate‐release cysteamine

## Abstract

Nephropathic cystinosis is a rare autosomal recessive lysosomal storage disease that is characterized by accumulation of cysteine and formation of crystals within cells of different organs and tissues causing systemic manifestations in childhood that include poor linear growth, ocular involvement, hypothyroidism, and progressive kidney disease. This study was a long‐term, prospective open‐label evaluation of twice‐daily delayed release (DR) cysteamine capsules in cystinosis patients <6 years of age who were naïve to any form of cysteamine treatment. Fifteen treatment‐naïve patients <6 years old (mean age 2.2 ± 1.0 years, 53% male, 73% White) were enrolled and treated with DR‐cysteamine capsules for up to 18 months. Patients had clinically meaningful decreases in WBC cysteine concentration during treatment (3.2 ± 3.0 nmol ½ cystine/mg protein at Day 1 to 0.8 ± 0.8 nmol ½ cystine/mg protein at study exit), and anthropometric data improvements were consistently observed in height, weight and body surface area. Additionally, estimated glomerular filtration rate increased from 55.93 ± 22.43 ml/min/1.73 m^2^ at baseline to 63.79 ± 21.44 ml/min/1.73 m^2^ at study exit. Pharmacokinetic/Pharmacodynamic results support the use of the same starting, escalation, and maintenance doses according to body surface for children aged <6 years that are currently recommended in adults and older children. All patients experienced ≥1 adverse event(s) with vomiting (80%) and upper respiratory tract infection (53%) most frequently reported. Based on our study, patients <6 years of age with nephropathic cystinosis without prior treatment can safely and effectively initiate treatment with DR‐cysteamine, a delayed‐release form of cysteamine bitartrate that can be given every 12 h.

**Synopsis**

Initial treatment of patients <6 years of age with nephropathic cystinosis can be safely and effectively started with DR‐CYS, a delayed‐release form of cysteamine bitartrate that can be given every 12 h.

## 1 INTRODUCTION

Nephropathic cystinosis (NC) (OMIM 219800; 219900) is a rare, autosomal recessive lysosomal storage disease caused by mutations in the *CTNS* gene that is estimated to affect 1 of every 100 000–200 000 live births.^1^ It is characterized by accumulation of cystine and formation of cystine crystals within cells of different organs and tissues, especially the kidney, leading to end‐stage renal disease by the end of the first decade of life if untreated.

Nephropathic cystinosis symptoms most commonly appear within the first year of life and present with generalized proximal tubular damage (Fanconi syndrome) and clinical signs of polyuria, polydipsia, and failure to thrive.^2^, ^3^, ^4^ Beyond kidney damage, the systemic manifestations that occur in childhood include poor linear growth,^5^ ocular involvement (clinically manifested by photophobia), and hypothyroidism.^2^, ^3^ Additionally, during the second or third decade of life patients may develop *diabetes mellitus*, hepatic involvement, bone disease, progressive muscle weakness, and central nervous system involvement, among other organ dysfunctions.^2^, ^6^

Even though new therapeutic options have been studied,^7^, ^8^ the current best available therapy involves depletion of intracellular cystine with cysteamine bitartrate, a cystine‐depleting antioxidant agent,^6^, ^9^, ^10^, ^11^, ^12^, ^13^ to prevent or delay organ pathology.^3^, ^6^ Cysteamine reacts within lysosomes to convert the accumulated cystine into cysteine & cysteine‐cysteamine mixed disulfide, both of which can exit the lysosome and thus lower the cystine content of cells in patients with cystinosis.^6^

The immediate‐release formulation of cysteamine bitartrate (IR‐CYS) requires a frequent administration of every 6 h, which makes routine nighttime dosing mandatory. This dosing regimen imposes chronic interruptions on sleep, which is likely to negatively affect quality of life for both patient and caregiver. Indeed, compliance to the round‐the‐clock 6 h dosing requirement has been found to be only about 23% and 17% of patients have admitted to only take the medication during the day.^14^ This lack of compliance is deleterious as cystine concentrations quickly increase if medication dosing is delayed even by a few hours.^14^, ^15^, ^16^

The delayed‐release formulation of cysteamine (DR‐CYS) is formulated with cysteamine bitartrate surrounded by an acid‐resistant enteric coating, which allows the cysteamine bitartrate to pass through the acidic stomach to the alkaline environment of the small intestine.^17^, ^18^, ^19^, ^20^ This formulation allows a gradual release of cysteamine and provides 12 h of continuous cystine control extending the duration of effective treatment and resulting in better adherence, improved quality of life, and disease control.^17^, ^18^, ^19^, ^20^, ^21^ Equivalence in control of WBC cystine concentration has been previously demonstrated between IR‐CYS and DR‐CYS in a study of 43 patients (average 12 years of age) when IR‐CYS dosed every 6 h was substituted with DR‐CYS that was administered every 12 h.^22^

Early diagnosis of NC and initiation of cystine depleting therapy (CDT) at diagnosis, as well as strict compliance to therapy, has been shown to significantly delay progression to end stage renal disease, improve growth, decrease the frequency and severity of extrarenal complications, and is associated with extended life expectancy.^23^ Therefore, early diagnosis of cystinosis and initiation of an unwavering life‐long treatment are essential for preventing end‐organ damage and improving the overall prognosis.

The primary objective of this study was to evaluate the safety and effectiveness of long‐term repeat dosing of DR‐CYS on white blood cell (WBC) cystine concentrations in nephropathic cystinosis patients <6 years of age who were naïve to cysteamine treatment. The study also evaluated a new treatment initiation and titration methodology designed to maximize tolerability in treatment‐naïve children under 6 years of age.

## 2 METHODS

### 2.1 Trial design

This study was designed as a long‐term, prospective open‐label evaluation of twice‐daily DR‐CYS capsules in cystinosis patients <6 years of age who were naïve to any form of cysteamine treatment (ClinicalTrials.gov identifier: NCT01744782). This study was approved by The Ann & Robert H. Lurie Children's Hospital of Chicago Institutional Review Board (2013‐15137) and by the Hospital das Clínicas, University of São Paulo Review Board (n°413.101). Informed consent was obtained from all parents/caregivers for being included in the study.

The inclusion criteria consisted of a documented diagnosis of cystinosis with no clinically significant change in liver function tests (i.e., 1.5 times upper limit of normal (ULN) for alanine aminotransferase (ALT) and aspartate aminotransferase (AST), and/or 1.5 times ULN for total bilirubin) within 6 months prior to screening. No clinically significant change in renal function (ie, estimated glomerular filtration rate [eGFR] within 6 months prior to screening and an eGFR >20 ml/min/1.73 m^2^). Required hemoglobin level was >10 g/dl at screening. Patients were excluded for any of the following: history of active inflammatory bowel disease or prior resection of the small intestine, heart disease, or active bleeding disorder within 90 days prior to screening, malignant disease within 2 years prior to screening, kidney transplant, or using dialysis at time of trial.

Patients were enrolled sequentially at each study site and paired Pharmacokinetic/Pharmacodynamic (PK/PD) sampling times were determined by randomization via an electronic Code of Federal Regulations (e‐CFR) database. DR‐CYS capsules were administered twice daily, orally or via gastrostomy tube (G‐tube) in patients with an established G‐tube (n = 3), after a 2‐h fast. The starting dose was one‐quarter of the targeted maintenance dose based on age, weight, and body surface area (BSA). The dose was gradually escalated, in 10% increments every 2 weeks, based on monitoring of mixed leukocyte WBC cystine levels measured 30 min after the morning dose and collected bi‐monthly (every 2 weeks), until the patient's WBC cystine level was <1 nmol ½ cystine/mg protein. The recommended targeted maintenance dose for children up to 6 years old was 1 g/m^2^/day, given every 12 h in two divided doses. A more gradual dose escalation or a dose reduction could have been considered for tolerability reasons.

Caregivers were instructed to try to avoid giving food to patients for 2 h before and at least 30 min after DR‐cysteamine administration. If patients could not comply with this recommendation, they were instructed to give only a small amount of food around the time of DR‐cysteamine dosing (from 1 h before to 1 h afterward) and importantly, withhold dairy products during this same time window.

DR‐CYS capsules were either swallowed whole or administered by sprinkling the capsule contents in an acceptable food such as an acidic media such as fruit juice and crushed fruit and not water, dairy or high fat foods, and taken orally or via G‐tube. It was recommended that the patient take the study drug with the same type of food or liquid consistently throughout the study. Treatment duration was at least 12 months.

All patients were asked about concomitant medications at study entry and at each study visit and asked to stop gastric acid‐reducing medications at least 12 h before receiving their first dose of DR‐CYS until study termination. In cases of intolerable gastric upset, gastric acid‐reducing medications were allowed, at the discretion of the Investigator. Growth hormone was not administered to any study patient; diet was ad‐lib without any restrictions or required supplementation. The management of these patients included sodium, potassium, chloride, magnesium, and alkali supplementation for hydro‐electrolyte and metabolic balance; vitamin D and thyroid hormone reposition if necessary. No other intervention was made.

Initially, the study included any patient with untreated cystinosis, but after enrolling four patients, two >6 years old (9 and 22 years old) and two <6 years old, the protocol was amended to include only patients <6 years old to satisfy a Food & Drug Administration (FDA) post marketing commitment. The data presented in this manuscript are focused on the 15 patients <6 years of age.

### 2.2 Outcomes and assessments

Anthropometric and safety parameters as well as clinical laboratory tests, physical examinations, and vital signs (systolic/diastolic blood pressure, heart rate, respiratory rate, and body temperature), and 12‐lead electrocardiograms (ECGs) were evaluated at each study visit, starting on Day 1, and thereafter at 6 bi‐monthly consecutive study visits, at least ≥3 quarterly visits, and a final exit visit. Blood was collected 30 min after the morning DR‐CYS dose at each visit and a subset of patients (n = 12) underwent frequent sampling at Month 6 for PD/PK parameter analysis.

#### 2.2.1 Anthropometric parameters

Body height was measured using a calibrated stadiometer or in the case of infants, body length was measured from the top of the head to the bottom of one heel. Body mass index (BMI) and BSA were calculated at each study visit.

#### 2.2.2 Pharmacodynamics

WBC cystine levels were measured via blood collection 30 min post morning DR‐CYS dose at Day 1, bi‐monthly, and exit visits, which corresponds to the steady‐state cysteamine‐trough concentration. At Month 6, WBC cystine levels were measured at three time points; 0 h (predose) and two randomized sampling times after the morning DR‐CYS dose that coincided with the PK sample collection. The WBC cystine concentrations were monitored using the mixed leukocyte method, which has a treatment target of <1.0 nmol ½ cystine/mg protein and determined using high‐performance liquid chromatography‐electrospray ionization tandem mass spectrometry (LC‐ESI‐MS/MS).^24^, ^25^

#### 2.2.3 Pharmacokinetics

Steady‐state cysteamine plasma concentration profiles were determined by blood collection 30 min after the morning DR‐CYS dose for Day 1 and bi‐monthly, quarterly and study exit visits. The Month 6 visit included collection of samples at 9 time points; 0 (predose), 30 min, 2, 3, 4, 6, 8, 10, and 12 h after the morning DR‐CYS dose. Plasma cysteamine concentration was determined using methods employing hydrophilic interaction liquid chromatography (HILC) high‐pressure liquid chromatography (HPLC) tandem mass spectrometry (HPLC‐MS/MS).^24^, ^25^

#### 2.2.4 Safety

Adverse events (AEs) and serious adverse events (SAEs) were coded using the Medical Dictionary for Regulatory Activities (MedDRA) version 16.1. SAEs were categorized from Grade 1 (mild AE) to Grade 5 (death related to AE) using the National Institutes of Health (NIH) Common Terminology Criteria for Adverse Events (CTCAE) Version 3.0.^26^

### 2.3 Statistical analysis

The PD and anthropometric analysis population included all patients who received at least 1 dose of DR‐CYS and who had at least one WBC cystine level recorded. The PK population included all patients who received at least one dose of DR‐CYS and had available PK data. The safety population included all patients who received at least one dose of DR‐CYS.

#### 2.3.1 Anthropometric parameters

Growth data (standing height, weight, BMI, and BSA) were summarized using descriptive statistics (number of patients, mean, median, standard deviation [SD], minimum, and maximum). Summaries were provided for the observed value, change from baseline, and percentage change from baseline. Additionally, the percentile and *Z*‐score (SD relative to a reference population) for a patient's gender and age were summarized descriptively at each study evaluation. The percentile and *Z*‐score were based on the Center for Disease Control and Prevention (CDC) growth data for the general population.^27^

#### 2.3.2 Kidney function parameters

Estimated GFR was determined using the creatinine‐based “Bedside Schwartz” equation, appropriate for children 1–18 years old,^28^, ^29^ as follows:

$\text{eGFR}=0.413*\left[\text{Height}\;\left(\mathrm{cm}\right)/\text{serum creatinine}\;\left(\mathrm{mg}/\mathrm{dl}\right)\right]$

#### 2.3.3 Pharmacodynamics

The WBC cystine levels were summarized using descriptive statistics (number of patients, mean, geometric mean [GM], coefficient of variation [CV], median, minimum, and maximum; or number of patients, mean, median, SD, minimum, and maximum). Summaries were provided for the observed value, change from baseline to Month 6, percentage change from baseline to Month 6, and proportion of patients who reached a WBC cystine level <1.0 nmol ½ cystine/mg protein at each visit.

#### 2.3.4 Pharmacokinetics

A noncompartmental analysis (NCA) was conducted using the frequently sampled PK data obtained at Month 6. Obtained PK parameters were characterized using dose, patient demographic characteristics, and study drug administration route. The correlation between obtained PK parameters and PD responses (WBC cystine concentration) at predose were investigated. The plasma PK parameters for cysteamine were estimated. *C*_max_ and *T*_max_ were taken directly from the data. The results of the current analyses were compared to the reported data of DR‐CYS in older children and healthy adults.

One‐way analysis of variance was used to compare between multiple groups, while the unpaired Student's *t*‐test (two‐tailed) was used to compare two groups. A *p*‐value of less than 0.05 was considered statistically significant. The correlations between PK parameters by NCA and patient demographics or PD response (WBC cystine concentration) were evaluated by linear regression analysis using GraphPad Prism (version 7.02, GraphPad Software Inc.).

#### 2.3.5 Safety

AEs were coded using the Medical Dictionary for Regulatory Activities (MedRA) version 16.1, and AE severity was assessed by the Investigator using CTCAE, Version 3.0. The actual value and change from baseline were summarized at all visits using descriptive statistics for each clinical laboratory parameter.

## 3 RESULTS

Seventeen patients were initially enrolled and treated; however, the focus of this study was on the 15 patients <6 years old. Therefore, the results presented are related to this group.

The diagnosis of NC was based on Fanconi syndrome associated with corneal cystine crystals in all 15 patients. Three patients had WBC cystine levels ≤1 nmol ½ cystine/mg protein at Day 1 of the study (30‐min after the drug administration). Notably, each of these three patients had one or more elevations of WBC cystine at later time points during the study period, consistent with a diagnosis of NC. Furthermore, genetic testing was performed in some of the study patients, including those three patients, and confirmed their NC diagnoses (Table 1).

**TABLE 1:** Patient demographics and study inclusion criteria

				Study inclusion criterion			
	Age at study Day 1 (y)	Gender	Race	Elevated WBC cystine (>1 nmol ½ cystine/mg protein)	*CTNS* gene mutation allele 1/allele 2	Detection of corneal cystine crystal deposits	Detection of cystine crystal deposits in bone marrow aspirates
1	1.618	F	White	3.830	NP	Positive	NP
2	1.095	F	White	2.440	NP	Positive	NP
3	2.418	M	White	1.428	NP	Positive	NP
4	1.043	F	White	8.095	NP	Positive	NP
5	4.507	F	Black or African American	5.507	del 57 kb/del 57 kb	Positive	NP
6	1.777	M	White	1.784	c.C382T:p.Gln128X/c.C382T:p.Gln128X	Positive	NP
7	1.875	F	Black or African American	0.911	del 57 kb/del 57 kb	Positive	Positive
8	2.568	M	White	10.888	del 57 kb/del 57 kb	Positive	NP
9	2.316	M	White	2.669	del 57 kb/del 57 kb	Positive	NP
10	2.270	M	Black or African American	1.623	NP	Positive	NP
11	3.682	M	Black or African American	3.977	NP	Positive	NP
12	2.396	F	White	0.647	del 57 kb/del 57 kb	Positive	NP
13	3.075	M	White	1.629	del 57 kb/del 57 kb	Positive	NP
14	1.021	F	White	0.423	del 57 kb/c.C382T:p.Q128X	Positive	NP
15	1.413	M	White	1.714	del exons 2,3,4/del exons 2,3,4	Positive	NP

Abbreviations: F, female; M, male; NP, not performed.

The 15 patients had a mean ± SD age of 2.2 ± 1.0 years (range of 1.0‐4.5 years). Eight patients (53.3%) were male and 11 patients (73.3%) were racially White consistent with the racial prevalence of the disease. Table 1 shows individual demographic data of these 15 patients as well as the study inclusion criteria for each subject such as elevated WBC Cystine levels, presence of biallelic *CTNS* pathogenic variants, positive corneal cystine crystal deposits and/or in bone marrow aspirates.

Fourteen patients completed the study with at least 12 months of treatment and 10 patients completed at least 18 months of treatment (Table 2). Thirteen of the 14 patients achieved their highest total daily dosage of DR‐CYS following the 9‐month visit (9‐month visit for 8 subjects, 12‐month visit for 4 subjects, and 18‐month visit for 1 subject). Mean study drug exposure was 511.7 ± 158.1 days (median 575.0 days; range 17‐611 days). Table 2 shows the total initial and final dosage of DR‐CYS, via of administration and the duration of treatment for each patient.

**TABLE 2:** Cysteamine‐DR dosing, route of administration, and duration of treatment (n = 15)

Patient	Cysteamine‐DR total daily dose; initial (mg)	Cysteamine‐DR total daily dose; final (mg)	Cysteamine‐DR method of dosing (initial/final)	Treatment duration (months)
1	50	500	Intragastric/intragastric	21
2	50	400	Intragastric/intragastric	15
3	150	600	Intragastric/intragastric	13
4	50	350	Intragastric/intragastric	17
5	150	250	Dietary/dietary	20
6	150	250	Dietary/oral	20
7	150	300	Dietary/intragastric	20
8	100	350	Oral/oral	20
9	150	600	Dietary/oral	20
10	100	450	Dietary/oral	20
11	100	Received 1 dose	Dietary	0.5
12	300	450	Dietary/oral	19
13	200	450	Dietary/oral	19
14	100	200	Dietary/intragastric	19
15	150	300	Oral/oral	14

All patients (100%) reported using at least one concomitant medication, which was started or continued after the first dose of study drug. The most frequently used concomitant medications were potassium chloride, phosphorus, calcitriol, and sodium bicarbonate (>70% of patients each); ferrous sulfate, ergocalciferol, omeprazole, and ranitidine (>40% of patients each); and sodium chloride (electrolyte solution), levothyroxine, colecalciferol, potassium citrate, magnesium sulfate, paracetamol, calcium carbonate, erythropoietin, sodium chloride (nasal preparation), ondansetron, and osmotan. The most frequent procedure was gastrostomy or reinsertion of G‐tube (three patients).

### 3.1 Pharmacodynamics

**FIGURE 1:**
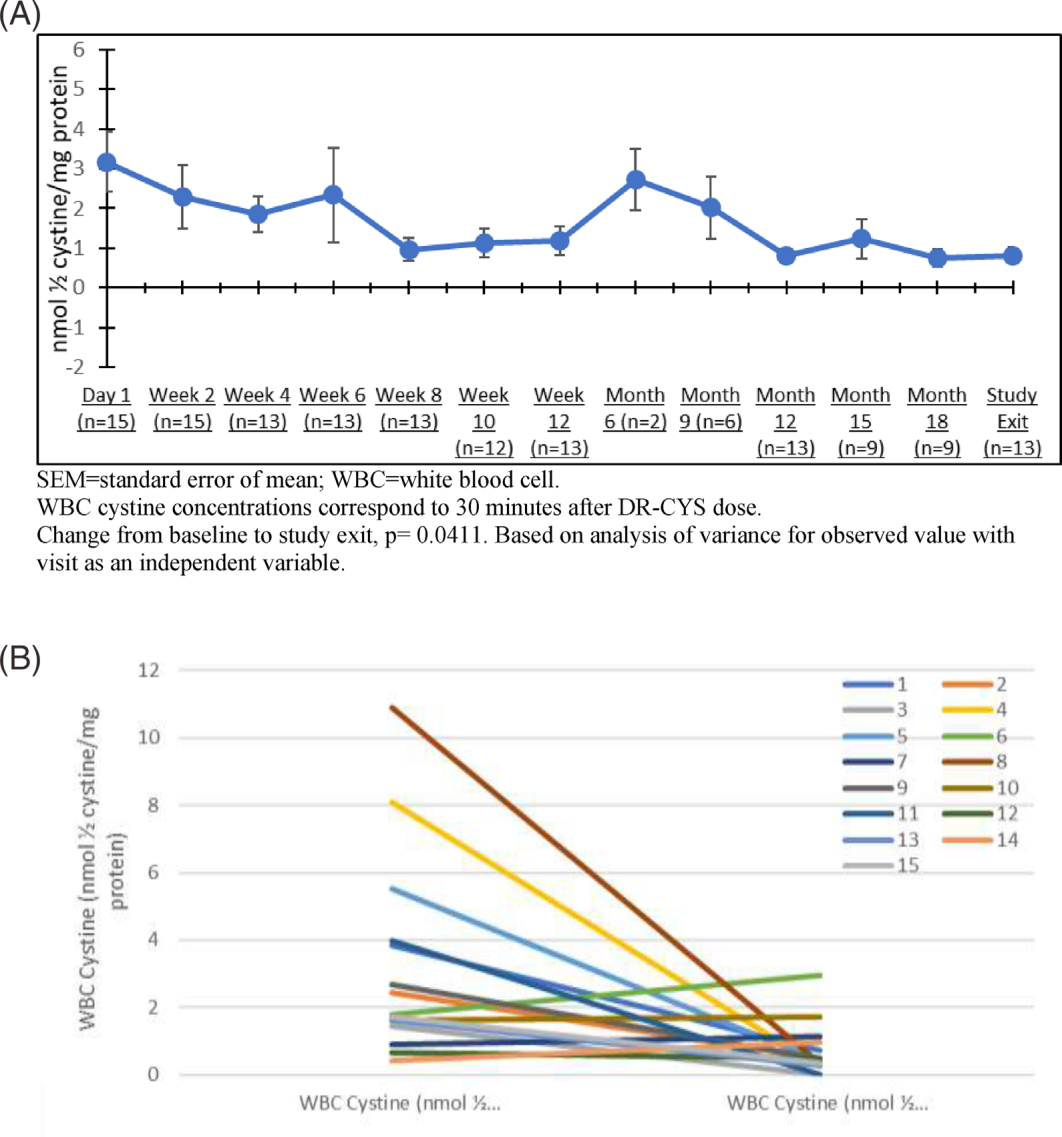
WBC cystine concentrations—mean (SEM)—at different time points over the study in all 15 patients (A) and the individual curves (B). (A) WBC cystine concentration, mean (SEM) at different time points over the treatment period. At Month 6 there were only samples from two patients. SEM, standard error of mean; WBC, white blood cell. WBC cystine concentrations correspond to 30 min after DR‐CYS dose. Change from baseline to study exit, *p* = 0.0411. Based on analysis of variance for observed value with visit as an independent variable. (B) Individual curves of WBC cystine concentration at the beginning of the study and at study exit. The number in the legend corresponds to the patient's number

There was a clinically meaningful decrease in mean (± SD) WBC cystine concentration over the treatment period, from a mean of 3.2 ± 3.0 nmol ½ cystine/mg protein at Day 1 to 0.8 ± 0.8 nmol ½ cystine/mg protein at study exit (Figure 1). The percentage of patients who reached WBC cystine <1.0 nmol ½ cystine/mg protein increased over time from 20.0% at baseline (3/15) to 76.9% at study exit (10/13). At the Month 6 assessment, which included multiple assessments, the predose mean was 1.6 ± 1.1 nmol ½ cystine/mg protein and decreased to 1.0 ± 1.0 nmol ½ cystine/mg protein by 4 h post dose. Post dose decreases ranged from −0.2 to −0.9 nmol ½ cystine/mg protein (−15.7 to −41.1%) across the assessment times except at 10 h, where there was a mean increase.

### 3.2 Anthropometric parameters

**FIGURE 2:**
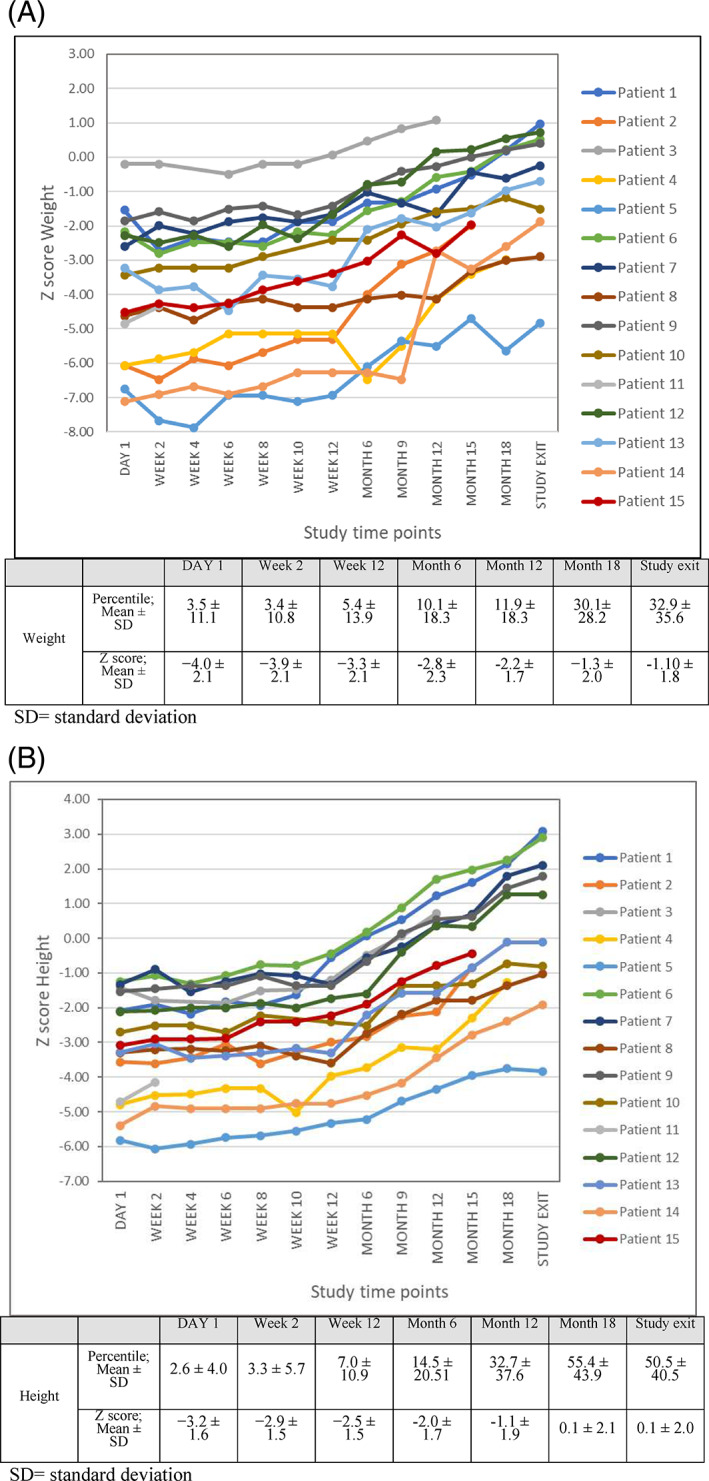
Evolution of *Z* score weight (A) and height over the study time points for each included patient (n = 15). (A) *Z* score weight for each patient over the study and mean (SD) at study time points. (B) *Z* score height for each patient over the study and mean (SD) at study time points. SD, standard deviation

There was a clinically meaningful increase in mean Z score standing height (−3.2 ± 1.6 on Day 1 to 0.1 ± 2.0 at study exit), *Z* score weight (−4.0 ± 2.1 on Day 1 to −1.10 ± 1.8 at study exit) and *Z* score BSA (−1.8 ± 1.1 on Day 1 to −1.3 ± 1.1 at study exit) over the course of the treatment period while mean *Z* score BMI remained relatively unchanged (−1.0 ± 1.1 on Day 1 to −1.2 ± 1.3). The anthropometric parameters are showed for each patient at the beginning of the study and at study exit in Table 3. Figure 2 shows individual curves for weight (A) and height (B) *Z* score.

**TABLE 3:** Anthropometric data of the study patients on Day 1 and at study exit for each patient (n = 15)

Patient	*Z* score height (m), Day 1	*Z* score height (m), study exit	*Z* score weight (kg), Day 1	*Z* score weight (kg), study exit	BMI (kg/m^2^), Day 1	BMI (kg/m^2^), study exit	BSA (m^2^), Day 1	BSA (m^2^), study exit
1	−2.09	3.08	−1.54	0,97	17.4	15.06	0.44	0.55
2	−3.56	−0.84	−6.07	−1.99	13.39	14.95	0.35	0.44
3	−1.42	0.72	−0.20	1.07	17.93	17.31	0.57	0.63
4	−4.79	−1.27	−6.07	−2.99	15.97	14.12	0.34	0.42
5	−5.82	−3.83	−6.75	−4.83	13.89	13.43	0.45	0.5
6	−1.24	2.9	−2.17	0.52	15.04	14.71	0.45	0.56
7	−1.33	2.11	−2.6	−0.25	15.67	14.14	0.43	0.51
8	−3.30	−1.03	−4.62	−2.9	13.78	13.15	0.44	0.49
9	−1.53	1.78	−1.86	0.40	15.92	14.74	0.53	0.62
10	−2.71	−0.81	−3.44	−1.51	14.84	14.85	0.46	0.53
11	−4.71	−4.15	−4.85	−4.34	14.13	NA	0.47	NA
12	−2.11	1.26	−2.28	0.72	15.86	15.72	0.48	0.61
13	−3.28	−0.12	−3.23	−0.70	15.69	14.62	0.52	0.62
14	−5.39	−1.91	−7.12	−1.88	15.12	16.57	0.33	0.44
15	−3.08	−0.44	−4.52	−1.96	14.04	14.66	0.39	0.46

### 3.3 Kidney function parameters

**FIGURE 3:**
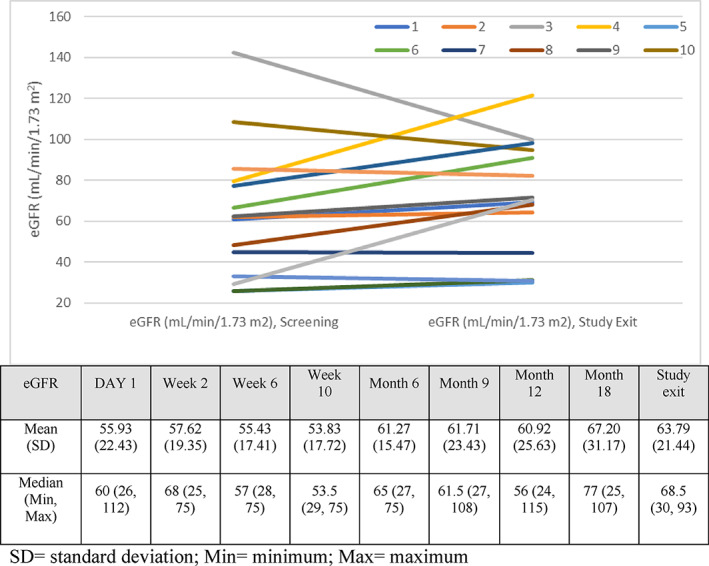
Estimated glomerular filtration rate (eGFR) for each patient (n = 15) at Day 1 of the study and study exit and mean (SD) and median (range) over the study time points. The number in the legend corresponds to the patient's number. Max, maximum; Min, minimum; SD, standard deviation;

**FIGURE 4:**
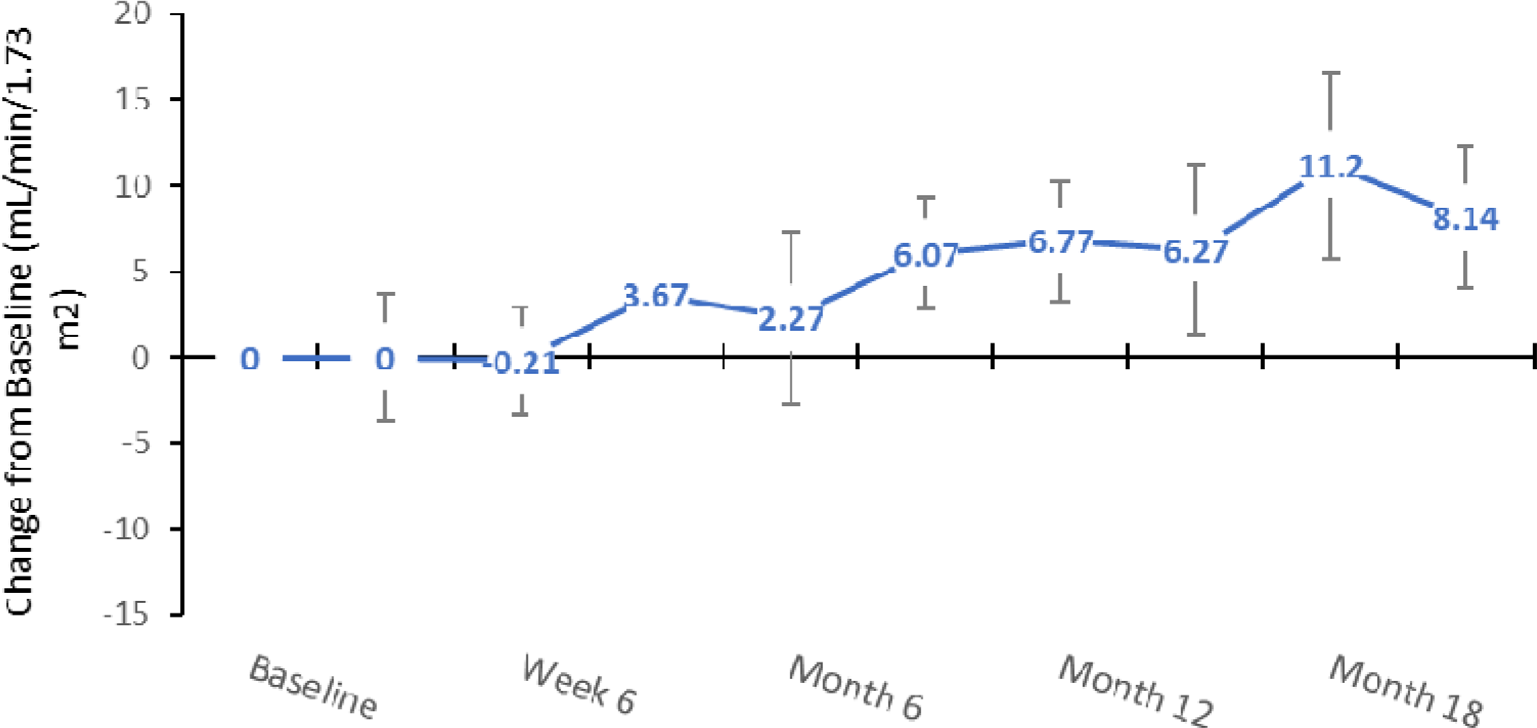
eGFR change from baseline (ml/min/1.73 m^2^); mean (SEM)

Mean eGFR increased over the course of the treatment period from 55.93 ± 22.43 ml/min/1.73 m^2^ (n = 15) at baseline to 63.79 ± 21.44 ml/min/1.73 m^2^ (n = 14) at study exit (mean change of 8.14 ± 15.48 ml/min/1.73 m^2^). Figure 3 shows the eGFR for each patient at Day 1 and study exit and mean (SD) and median (range) at different study time points. There was a significant improvement in eGFR as one can see in Figure 4.

### 3.4 Pharmacokinetics

**FIGURE 5:**
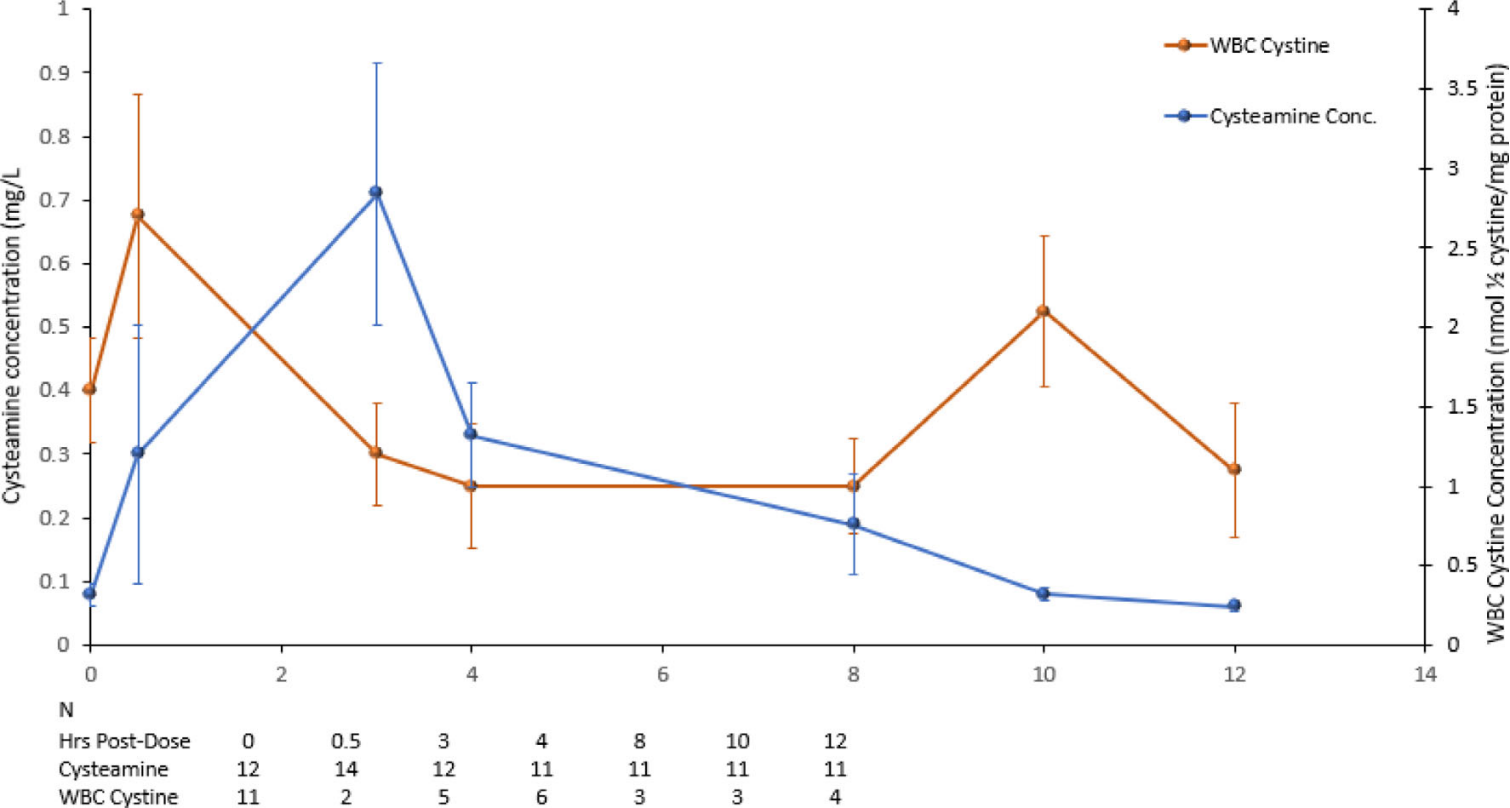
Cysteamine concentration and WBC cystine level at Month 6 (N = 14); mean (SEM)

The plasma cysteamine concentration‐time profiles for the 12 patients who participated in frequent sampling at Month 6 demonstrated a large interpatient variability in cysteamine PK with most patients exhibiting delayed absorption. Pooled plasma cysteamine concentration‐time profiles plotted with WBC cystine concentration at Month 6 is shown in Figure 5. After the administration of a single dose of DR‐CYS, peak concentrations of cysteamine were observed at 3 h postdose and returned to baseline concentrations by 12 h‐postdose. Overall, the WBC cystine content decreased when the plasma cysteamine concentration increased.

The relationship between individual PD data (WBC cystine concentration) at time 0 (predose) and obtained PK parameters at steady state showed association with dose and PK parameters. The *C*_max_ showed higher correlation (*R* ^2^ = .369) compared to dose and the other PK parameters but was not statistically significant.

Pharmacokinetic parameters estimated using NCA for the frequent sampling at Month 6 are summarized in (Table 4). A dose‐dependent increase in AUC_inf_ was observed (*R* ^2^ = .747, N = 10). However, for V/F (L) and CLss/F (L/min), significant associations with patient demographics (age, body weight, and BSA) were not observed. Overall, the pharmacokinetics in patients between the ages of 1 and 5 years of age is comparable with those in older children and adults.^21^

**TABLE 4:** Summary of cysteamine pharmacokinetic parameters at Month 6

	*T*_max_ (min)	*C*_max_ (mg/L)	AUC_last_ (min*mg/L)	AUC_inf_ (min*mg/L)	CLss/F (L/min)	V/F (L)	*t*_1/2_ (min)
n	11	11	11	10	11	10	10
Mean	199	1.26	206	231	0.69	241	270
SD	138	0.86	113	123	0.37	118	56
Median	120	1.11	180	211	0.57	234	282
Minimum	30	0.28	67	84	0.31	104	205
Maximum	480	2.92	398	423	1.53	488	396

*Note*: AUC_last_, area under the plasma concentration versus time curve, from time 0 to the time of the last measurable concentration (720 min); AUC_inf_, area under the plasma concentration versus time curve from time 0 to infinity; CLss/F, apparent total body clearance from plasma; *C*_max_, maximum observed plasma concentration; min, minutes; SD, standard deviation; *t*_1/2_ = apparent terminal elimination half‐life; *T*_max_ = time of maximum observed plasma concentration; V/F, apparent volume of distribution based on the terminal phase.

Comparisons of PK parameters by age (infants vs. children) and by route of study drug administration (oral, intragastric, and dietary [sprinkled in food]) were planned. However, analyses by age were not feasible because of the small sample sizes for infants (n = 2). It was difficult to draw definitive conclusions from analyses by route of administration, but overall no obvious differences in PK parameters between oral and G‐tube administration was observed. The routes of administration for each patient are included in Table 2.

### 3.5 Safety

The overall incidence of AEs was 100% in both the Titration period (n = 15) and the maintenance period (n = 14). The most frequently reported AEs (≥10% of patients) and the SAEs can be found in Table 5. There were no notable differences in AE incidence between the two periods except for cough (27% titration; 0% maintenance) and upper respiratory infection (13% titration; 50% maintenance). For most patients (≥93%), the onset of their first AE occurred in the first 90 days of the study.

**TABLE 5:** Adverse events observed during study period

	Titration	Maintenance	Overall
	(N = 15)	(N = 14)	(N = 15)
Number of patients with AEs	n (%)	n (%)	n (%)
Patients with Any AE	15 (100)	14 (100)	15 (100)
*AEs occurring in >10% of patients*			
Vomiting	8 (53.3)	7 (50.0)	12 (80.0)
Upper respiratory tract infection	2 (13.3)	7 (50)	8 (53.3)
Gastroenteritis	2 (13.3)	5 (35.7)	7 (46.7)
Diarrhea	4 (26.7)	3 (21.4)	5 (33.3)
Dehydration	1 (6.7)	3 (21.4)	4 (26.7)
Pyrexia	1 (6.7)	3 (21.4)	4 (26.7)
Cough	4 (26.7)	0	4 (26.7)
Rhinitis	3 (20.0)	0	3 (20.0)
Breath odor	3 (20.0)	0	3 (20.0)
Nausea	2 (13.3)	1 (7.1)	3 (20.0)
Electrolyte imbalance	0	2 (14.3)	2 (13.3)
Rhinorrhea	2 (13.3%)	0	2 (13.3)
Gastrostomy	0	2 (14.3)	2 (13.3)
Headache	0	2 (14.3)	2 (13.3)
Dermatitis diaper	0	2 (14.3)	2 (13.3)
*SAEs*			*n (%)*
Gastroenteritis			5 (33.3)
Dehydration			4 (26.7)
Vomiting			4 (26.7)
Electrolyte imbalance			2 (13.3)
Gastrostomy			2 (13.3)
Catheter site infection, clostridium difficile colitis, gastroenteritis viral, failure to thrive, hypernatremia, hypocalcemia, malnutrition, metabolic acidosis, abdominal distension, anaemia, cardiopulmonary failure, fanconi syndrome, and hypovolemic shock			1 (6.6%)

Twelve patients (80.0%) had at least one SAE for a total of 36 SAEs. All SAEs were considered not related to the study drug. One patient died on Day 17 of study drug treatment, during the titration period, the patient had four grade 5 SAEs: gastroenteritis, hypovolemic shock, cardiopulmonary failure, and Fanconi syndrome. The subject was hospitalized and died the next day. In the Investigator's assessment, the SAEs were likely related to the underlying cystinosis and not related to the study drug.

## 4 DISCUSSION

Nephropathic cystinosis is a rare systemic lysosomal storage disease in which dysfunctional cystinosin (a lysosomal membrane transport protein) leads to intralysosomal cystine accumulation in cells throughout the body.^2^, ^29^, ^30^ The natural history of this rare disease was transformed by the discovery that cysteamine could act as a cystine‐depleting agent and a partial antioxidant drug.^2^, ^6^, ^12^, ^13^, ^29^ Many studies have demonstrated the benefits of cysteamine's cystine‐depleting effects to postpone end stage renal disease and delay and mitigate progression of multiple extra‐renal manifestations of cystinosis. Initiating cysteamine treatment at diagnosis of cystinosis, ideally within the first year of life, is associated with favorable clinical outcomes, including slowing renal function deterioration, preventing hypothyroidism, and improving growth parameters.^3^, ^31^, ^32^ However, beyond the side effects observed with IR cysteamine, even patients under regular treatment generally have a persistent low stature, progressive renal dysfunction and systemic extra‐renal involvements.^33^, ^34^, ^35^ The necessity of administering IR cysteamine every 6 h requires a middle of the night dose, every night of the patient life. Compliance with IR‐CYS is a difficult challenge invariably affecting sleep nightly for patients and caregivers as well as being associated with poor disease control and less favorable health outcomes.^16^, ^36^, ^37^ DR‐CYS is formulated to be released in the small intestine instead of stomach which extends dosing to every 12 h and is likely to improve dosing adherence.^37^ Previous studies have demonstrated noninferiority of DR‐CYS in relation to IR‐CYS to treat NC patients switched from IR to DR‐CYS. Two years of study data with optimal maintenance of the disease under a controlled study protocol demonstrated that with DR‐CYS, there was preservation of kidney function, stable somatic growth and BMI, as well as improved quality of life for patients that switched to DR‐CYS from IR‐CYS, and these changes were maintained for the entire 24‐month study period.^17^, ^22^ The results from the current study demonstrate the importance of initiation of treatment with DR‐CYS capsules as soon as the diagnosis of cystinosis is confirmed in the pediatric population (<6 years), because of the critical importance of proper treatment in the preservation of renal function and anthropometric growth.^36^, ^38^, ^39^

In the current protocol, anthropometric data improvements were consistently observed in height, weight, and BSA. Improvements in growth have not been previously published as it relates to treatment in this patient population and possible contributing factors to our findings may have been the younger patient population and early initiation of treatment. The patients in this study were younger than other published studies, and as a result, were treated earlier and with the benefit of frequent monitoring and dose escalating titrations following careful management within a monitored clinical study. A previous study of 94 pediatric patients with a mean age of 46 months at study entry, showed that patients on IR‐CYS treatment maintained growth (ie, did not show increasing growth failure compared with normal scales) although growth velocity did not increase enough to allow patients to catch up to age norms for height.^40^ The BMI findings in this study were not unexpected as children who undergo cysteamine treatment for the management of nephropathic cystinosis grow, as evidenced in this study by increased height, weight, and BSA; however, clinical experience shows that these children tend to remain lean.^41^ In addition, plasma cysteamine concentrations in this study correlated with reductions in the measured WBC cystine concentrations demonstrating control of the damaging effects of intracellular cystine buildup.

This is the first prospective trial of cysteamine treatment using DR‐CYS to demonstrate an increase in eGFR from baseline to end of study in children <6 years of age. With the focus of our patient population being younger than most in published literature,^17^, ^36^, ^38^ the increase in eGFR over time may reflect the benefits of early treatment initiation in these very young children. The initiation of treatment at onset of diagnosis and closely monitored healthcare during the study may have facilitated the correction of metabolic derangements inherent to the disease and as a result, these patients experienced a more normalized growth and development of the kidneys. In a previous clinical study with DR‐CYS in an older patient population ranging from 6 to 26 years (mean age 12 years), mean estimates of renal function as measured by the eGFR, were maintained through the extension period of 60 months.^21^ An increase in eGFR in this patient population, as seen in the current study with a mean change of 8.14, is an encouraging result and supports the use of DR‐CYS for improving kidney health in this patient population.

PK/PD profiles were similar to those reported for older children and adults however, overall cysteamine exposure was lower than what has been reported in older children or adults,^22^ which is consistent with the lower average dose employed in this study. Furthermore, the WBC cystine content at certain time points (ie, 6 and 9 months) was higher than the target of <1.0 nmol ½ cystine/mg protein which is partially explained by the low number of patients at these time points and by the fact that DR‐CYS was not administered at a fixed dose, but individually titrated based on tolerability and response. From a clinical pharmacology perspective, the dose titration process could be shortened by using a higher starting dose or allowing larger than the 10% titration increments that were utilized in this study. Large inter‐patient variability was observed in the current study, which was a similar observation to a previous study of older children and adults.^22^ The PK results from this study support the use of the same starting, escalation, and maintenance doses according to body surface for children aged <6 years that are currently recommended in adults and older children.

Some limitations of this study include the small size of the studied population due to the rarity of the disorder and the open‐label, noncomparative nature of the study. In this regard, the addition of PK and PD analysis may have improved the reliability of the results by evaluating a correlation between physiologic and observed clinical effects.^39^ Even with a small sample as in this study, a normal distribution of the current study data was detected. This fact emphasizes the consistency of the study design in the evaluated variables of our study.

## 5 CONCLUSIONS

De novo treatment of young children with nephropathic cystinosis with DR‐CYS, the delayed‐release form of cysteamine bitartrate, was well tolerated and effective at achieving a reduction in WBC cysteine concentration and an improvement in growth parameters and eGFR in patients <6 years of age throughout the treatment period of 12 months. Based on our study, initial treatment of patients <6 years of age with nephropathic cystinosis can be safely and effectively started with DR‐CYS, a delayed‐release form of cysteamine bitartrate that can be given every 12 h.

## CONFLICT OF INTEREST

Craig B. Langman declares that he was consultant to Horizon Therapeutics plc during the time of this study. Juliana Caires Ferreira, Heather Price, and Kyleen D. Young declare no conflict of interest. Saba Sile and Gregg Checani are Horizon Therapeutics plc employees. Maria Helena Vaisbich has received grants for travel from Recordatti Rare Disease and has received speaker honorarium from Horizon Therapeutics and Recordatti Rare Disease during the time of this study.

## AUTHOR CONTRIBUTIONS

Maria Helena Vaisbich: recruitment of patients to the study, analysis, and interpretation of data. Drafted and extensively revised the article critically for important intellectual content. Juliana Caires de Oliveira Achili Ferreira, Heather Price, and Kyleen D. Young: provided clinical and biological data, revised the article. Saba Sile and Gregg Checani: statistical analysis and interpretation of data, revised the article. Craig B. Langman: conception and design of the study, recruitment of patients to the study, analysis, and interpretation of data. Drafted and extensively revised the article.

## ETHICS STATEMENT

All procedures followed were in accordance with the ethical standards of the responsible committee on human experimentation (institutional and national) and with the Helsinki Declaration of 1975, as revised in 2000 (5). It was approved by The Ann & Robert H. Lurie Children's Hospital of Chicago Institutional Review Board (2013‐15137) and by the Hospital das Clínicas, University of São Paulo Review Board (n°413.101). Prior to study participation the patient's parent/guardian received information about the protocol and signed the written informed consent.

## References

[1] Elmonem MA , Veys KR , Soliman NA , van Dyck M , van den Heuvel LP , Levtchenko E . Cystinosis: a review. Orphanet J Rare Dis. 2016;22(11):47. 10.1186/s13023-016-0426-y PMC4841061 27102039

[2] Goodyer P . The history of cystinosis: lessons for clinical management. Int J Nephrol. 2011;2011:929456. 10.4061/2011/929456 22013525 PMC3195959

[3] Ivanova E , De Leo MG , De Matteis MA , Levtchenko E . Cystinosis: clinical presentation, pathogenesis and treatment. Pediatr Endocrinol Rev Suppl. 2014;1:176‐184. 25345100

[4] Bäumner S , Weber LT . Nephropathic cystinosis: symptoms, treatment, and perspectives of a systemic disease. Front Pediatr. 2018;6:58. 29594088 10.3389/fped.2018.00058 PMC5861330

[5] Besouw M , Levtchenko E . Growth retardation in children with cystinosis. Minerva Pediatr. 2010;62:307‐314. 20467383

[6] Gahl WA , Thoene JG , Schneider JA . Cystinosis. N Engl J Med. 2002;347:111‐121. 12110740 10.1056/NEJMra020552

[7] Brasell EJ , Chu LL , Akpa MM , et al. The novel aminoglycoside, ELX‐02, permits CTNSW138X translational readthrough and restores lysosomal cystine efflux in cystinosis. PLoS One. 2019;14(12):e0223954. 31800572 10.1371/journal.pone.0223954 PMC6892560

[8] Elmonem MA , Veys K , Oliveira Arcolino F , et al. Allogenic HSCT transfers wild‐type cystinosin to nonhematological epithelial cells in cystinosis: first human report. Am J Transplant. 2018;18(11):2823‐2828. 30030899 10.1111/ajt.15029

[9] Langman CB . Oh cystinosin: let me count the ways. Kidney Int. 2019;96:275‐277. 31331465 10.1016/j.kint.2019.05.016

[10] Emma F , Nesterova G , Langman C , et al. Nephropathic cystinosis: an international consensus document. Nephrol Dial Transplant. 2014;29(Suppl 4):87‐94. 10.1093/ndt/gfu090 PMC4158338 25165189

[11] Langmam CB , Barshop BA , Deschenes G , et al. Controversies and research agenda in nephropathic cystinosis: conclusions from a “Kidney Disease: Improving Global Outcomes” (KDIGO) Controversies Conference. Kidney Int. 2016;89:1192‐1203. 27181776 10.1016/j.kint.2016.01.033

[12] Okamura DM , Bahrami NM , Ren S , Pasichnyk K , Williams JM , Gangoiti JA et al (2014) Cysteamine modulates oxidative stress and blocks myofibroblast activity in CKD. J Am Soc Nephrol Jan;25(1):43–54. 24009239 10.1681/ASN.2012090962 PMC3871767

[13] Wilmer MJ , Kluijtmans LA , van der Velden TJ , et al. Cysteamine restores glutathione redox status in cultured cystinotic proximal tubular epithelial cells. Biochim Biophys Acta. 2011;1812(6):643‐651. 21371554 10.1016/j.bbadis.2011.02.010

[14] Levtchenko EN , van Dael CM , de Graaf‐Hess AC , et al. Strict cysteamine dose regimen is required to prevent nocturnal cystine accumulation in cystinosis. Pediatr Nephrol. 2006;21(1):110‐113. 16252107 10.1007/s00467-005-2052-0

[15] Kleta R , Bernardini I , Ueda M , et al. Long‐term follow‐up of well‐treated nephropathic cystinosis patients. J Pediatr. 2004;145(4):555‐560. 15480385 10.1016/j.jpeds.2004.03.056

[16] Ariceta G , Lara E , Camacho JA , et al. Cysteamine (Cystagon) adherence in patients with cystinosis in Spain: successful in children and a challenge in adolescents and adults. Nephrol Dial Transplant. 2015;30(3):475‐480. 25348508 10.1093/ndt/gfu329 PMC4339688

[17] Langman CB , Greenbaum LA , Grimm P , et al. Quality of life is improved and kidney function preserved in patients with nephropathic cystinosis treated for 2 years with delayed‐release cysteamine bitartrate. J Pediatr. 2014;165(528–33):e1. 10.1016/j.jpeds.2014.05.013 PMC4181581 24948347

[18] Veys KR , Besouw MT , Pinxten AM , van Dyck M , Casteels I , Levtchenko EN . Cystinosis: a new perspective. Acta Clin Belg. 2016;71(3):131‐137. 25560059 10.1179/2295333714Y.0000000113

[19] Dohil R , Fidler M , Gangoiti JA , Kaskel F , Schneider JA , Barshop BA . Twice‐daily cysteamine bitartrate therapy for children with cystinosis. J Pediatr. 2010;156(1):71‐75. 19775699 10.1016/j.jpeds.2009.07.016

[20] Dohil R , Rioux P . Pharmacokinetic studies of cysteamine bitartrate delayed‐release. Clin Pharmacol Drug Dev. 2013;2(2):178‐185. 27121672 10.1002/cpdd.12

[21] *Procysbi® (cysteamine bitartrate) prescribing information*. Horizon Pharma USA, Inc.; 2017.

[22] Langman CB , Greenbaum LA , Sarwal M , et al. A randomized controlled crossover trial with delayed‐release cysteamine bitartrate in nephropathic cystinosis: effectiveness on white blood cell cystine levels and comparison of safety. Clin J Am Soc Nephrol. 2012;7:1112‐1120. 22554716 10.2215/CJN.12321211 PMC3386675

[23] Ariceta G , Giordano V , Santos F . Effects of long‐term cysteamine treatment in patients with cystinosis. Pediatr Nephrol. 2019;34(4):571‐578. 29260317 10.1007/s00467-017-3856-4 PMC6394685

[24] Dalton N , Turner C . Leukocyte cystine: the key measurement. J Inherit Metab Dis. 2005;28(6):1206‐1207.

[25] Dohil R , Fidler M , Barshop BA , et al. Understanding intestinal cysteamine bitartrate absorption. J Pediatr. 2006;148(6):764‐769. 16769383 10.1016/j.jpeds.2006.01.050

[26] Cancer therapy evaluation program, common terminology criteria for adverse events, version 3.0, DCTD, NCI, NIH, DHHS. Accessed March 31, 2003 (http://ctep.cancer.gov). Publish Date: August 9, 2006.

[27] SAS program (ages 0 to <20 years). Centers for Disease Control and Prevention. Centers for Disease Control and Prevention. Accessed 26 February 2019, www.cdc.gov/nccdphp/dnpao/growthcharts/resources/sas.htm.

[28] Schwartz GJ , Muñoz A , Schneider MF , et al. New equations to estimate GFR in children with CKD. J Am Soc Nephrol. 2009;20(3):629‐637. 19158356 10.1681/ASN.2008030287 PMC2653687

[29] Wilmer MJ , Emma F , Levtchenko EN . The pathogenesis of cystinosis: mechanisms beyond cystine accumulation. Am J Physiol. 2010;299(5):F905‐F916. 10.1152/ajprenal.00318.2010 20826575

[30] Kalatzis V , Nevo N , Cherqui S , Gasnier B , Antignac C . Molecular pathogenesis of cystinosis: effect of CTNS mutations on the transport activity and subcellular localization of cystinosin. Hum Mol Genet. 2004;13:1361‐1371. 15128704 10.1093/hmg/ddh152

[31] Pottel H , Vrydags N , Mahieu B , Vandewynckele E , Croes K , Martens F . Establishing age/sex related serum creatinine reference intervals from hospital laboratory data based on different statistical methods. Clin Chim Acta. 2008;396(1–2):49‐55. doi:10.1016/j.cca.2008.06.017 18621041

[32] Schwartz GJ , Haycock GB , Edelmann CM Jr , Spitzer A . A simple estimate of glomerular filtration rate in children derived from body length and plasma creatinine. Pediatrics. 1976;58(2):259‐263. 951142

[33] Brodin‐Sartorius A , Tête MJ , Niaudet P , et al. Cysteamine therapy delays the progression of nephropathic cystinosis in late adolescents and adults. Kidney Int. 2012;81(2):179‐189. 21900880 10.1038/ki.2011.277

[34] Greco M , Brugnara M , Zaffanello M , Taranta A , Pastore A , Emma F . Long‐term outcome of nephropathic cystinosis: a 20‐year single‐center experience. Pediatr Nephrol. 2010;25:2459‐2467. 20803298 10.1007/s00467-010-1641-8

[35] Vaisbich MH , Koch VH . Report of a Brazilian multicenter study on nephropathic cystinosis. Nephron Clin Pract. 2010;114:c12‐c18. 19816039 10.1159/000245065

[36] Nesterova G , Williams C , Bernardini I , Gahl WA . Cystinosis: renal glomerular and tubular function in relation to compliance with cystine‐depleting therapy. Pediatr Nephrol. 2015;30(6):945‐951. 25526929 10.1007/s00467-014-3018-x

[37] Besouw M , Tangerman A , Cornelissen E , Rioux P , Levtchenko E . Halitosis in cystinosis patients after administration of immediate‐release cysteamine bitartrate compared to delayed‐release cysteamine bitartrate. Mol Genet Metab. 2012;107(1–2):234‐236. 22832073 10.1016/j.ymgme.2012.06.017

[38] Markello TC , Bernardini IM , Gahl WA . Improved renal function in children with cystinosis treated with cysteamine. N Engl J Med. 1993;328(16):1157‐1162. 8455682 10.1056/NEJM199304223281604

[39] Kimonis VE , Troendle J , Rose SR , Yang ML , Markello TC , Gahl WA . Effects of early cysteamine therapy on thyroid function and growth in nephropathic cystinosis. J Clin Endocrinol Metab. 1995;80(11):3257‐3261. 7593434 10.1210/jcem.80.11.7593434

[40] *Cystagon (Cysteamine Bitartrate Capsules) Prescribing Information*. Mylan Pharmaceuticals Inc.; 2019.

[41] Besouw MT , Van Dyck M , Francois I , Van Hoyweghen E , Levtchenko EN . Detailed studies of growth hormone secretion in cystinosis patients. Pediatr Nephrol. 2012;27(11):2123‐2127. 22664570 10.1007/s00467-012-2213-x

